# Extrauterine growth restriction in very low birth weight infants according to different growth charts: A retrospective 10 years observational study

**DOI:** 10.1371/journal.pone.0283367

**Published:** 2023-04-20

**Authors:** Meta Starc, Manuela Giangreco, Giacomo Centomo, Laura Travan, Jenny Bua

**Affiliations:** 1 Institute for Maternal and Child Health - IRCCS “Burlo Garofolo”, Neonatal Intensive Care Unit, Trieste, Italy; 2 Institute for Maternal and Child Health - IRCCS “Burlo Garofolo”, Clinical Epidemiology and Public Health Research Unit, Trieste, Italy; University of Lausanne: Universite de Lausanne, SWITZERLAND

## Abstract

**Background:**

Extrauterine growth restriction (EUGR) is common among very low birth weight (VLBW) infants and associated with poor neurodevelopmental outcomes. There are two types of EUGR definitions (cross-sectional and longitudinal) and many growth charts for monitoring postnatal growth. Aims of our study were 1) to compare the rate of small for gestational rate (SGA) and EUGR in a population of VLBW infants, both according to different growth charts (Fenton, INeS charts and Intergrowth-21) and different definitions; 2) to identify risk factors for EUGR.

**Methods:**

This is a single centre retrospective observational study, including all VLBW infants born between January 2009 and December 2018. Anthropometric measures were obtained at birth and at discharge and presented as z-scores according to three growth charts (Fenton, INeS charts, Intergrowth-21). Maternal, clinical and nutritional data were retrieved from clinical records.

**Results:**

228 VLBW were included. Percentage of SGA did not change significantly according to the three different growth charts (Fenton 22.4%, INeS charts 22.8%, Intergrowth 28.2%, p 0.27). Prevalence of EUGR was significantly higher when INeS and Fenton charts were used, compared to Intergrowth charts regardless of EUGR-definition (*cross sectional*-EUGR: Fenton 33.5%, INeS charts 40.9%, Intergrowth-21 23.8%, p 0.001; *longitudinal*-EUGR (loss of 1SDS): Fenton 15%, INeS charts 20.4%, Intergrowth 4%, p <0.001). In our population a longer time to reach 100 ml/kg/day of enteral feeding increased of 18% the risk of longitudinal EUGR. Late onset sepsis and retinopathy of prematurity were associated with an increased risk of longitudinal EUGR, although not significantly, while having a preeclamptic mother was associated with a reduced risk.

**Conclusions:**

We confirmed a wide variability of EUGR rates when using different charts and definitions, highlighting that Intergrowth-21 charts identify less EUGR when compared to INeS and Fenton charts. Standardized criteria for defining EUGR are warranted in order to facilitate comparisons between studies and to improve the nutritional management of VLBW infants.

## Introduction

Adequate growth of preterm infants remains a challenge for neonatologists. Extrauterine growth restriction (EUGR), defined as poor growth during hospitalization, is common both in preterm and very low birth weight babies (VLBW). Many factors are known to influence EUGR such as periods of inadequate nutrition, feeding intolerance and a range of morbidities associated with preterm birth (respiratory distress, patent ductus arteriosus, anaemia, late onset sepsis, bronchopulmonary dysplasia) [1]. Moreover, EUGR has been associated with later impaired neurodevelopment [2]. Optimizing nutrition and postnatal growth is, therefore, a fundamental component of the management of preterm infants and an important health outcome measure in Neonatal Intensive Care Units (NICUs) [3]. However, results of existing studies on EUGR are difficult to compare, as there is a wide variability in the charts used for growth assessment and a lack of a consistent definition of EUGR.

Current EUGR definitions can be classified in (1) cross-sectional as weight at a given time <10^th^ centile, independently of birthweight or (2) longitudinal as weight loss between birth and a given time with different standard deviation thresholds (ΔSDS) [4]. Moreover, growth charts have been developed with different approaches: (1) cross-sectional, based on the size at birth of premature infants in population-based surveys, (2) longitudinal studies of postnatal growth of cohorts of preterm infants, and (3) estimates of fetal weight from ultrasonography scans [5].

In cross-sectional charts, assessment is based on intrauterine growth standards and does not reflect the adaptation of premature infants to extrauterine life. The most used cross-sectional growth chart is Fenton’s [6], derived from a meta-analysis of 6 large population-based surveys from developed countries including almost four million births. The cross-sectional Italian Neonatal Study (INeS) charts were developed between 2005 and 2007 with a nationwide prospective study involving 22,087 girls and 23,375 boys with both parents of Italian origin, born between 23 and 42 gestational weeks [7]. This study is part of the Fenton metanalysis [6].

The most recently developed charts with a longitudinal approach are the Intergrowth-21 [8]. The Intergrowth 21^st^ Project was a prospective, longitudinal, multicentre, multi-ethnic study with strict selection criteria and it describes optimal rather than average growth of preterm infants.

Previous studies showed that the rate of neonates either classified as small for gestational age (SGA) or as having EUGR changes remarkably when assessed with different charts [9–12], and the prevalence of EUGR is strongly influenced by its different definitions [11].

The primary aim of the present study was to describe the differences of prevalence of SGA and EUGR among very low birth weight (VLBW) infants born in an Italian referral hospital, according to the three growth charts most commonly used in Italy (Fenton, INeS and Intergrowth) and according to EUGR different definitions (*cross-sectional* versus *longitudinal*). Secondary aim was to identify maternal, neonatal and nutritional factors associated with EUGR in our VLBW population.

## Material and methods

Ours is a retrospective observational cross-sectional time-series study conducted at the Institute of Maternal and Child Health, IRCCS Burlo Garofolo, Trieste (Italy), a public tertiary level university hospital with approximately 1600 births annually. The study was approved by our Institutional Board Review (RC 09/2020) which waived consent for retrospective data access from medical records. Data were fully anonymised before data analysis. Our study population included infants with a birthweight ≤1500 g born between the 1^st^ of January 2009 and the 31^st^ December 2018 and admitted within 72 hours of birth to our NICU. Infants who were transferred to other hospitals before 33 weeks of postmenstrual age, with chromosomal abnormalities and/or major congenital abnormalities were excluded. In the study period there were no changes in the NICU nutritional protocols.

Clinical and growth data were retrospectively collected from hospital records. The anthropometric measures of VLBW infants were captured at birth and discharge. The discharge criteria are as follows: cardiorespiratory stability in room air, good and regular growth in the last week and feeding autonomy since at least the last 48–72 hours. Infants who had been transferred to other hospitals after 33 weeks of postmenstrual age have also been included in our analysis. The discharge criteria in this case are the same, except for feeding autonomy. Birth and growth data were presented as centiles (Standard Deviation Score or z-score) according to the three reference standards (Fenton, INeS charts and Intergrowth-21). For the Intergrowth 21^st^ Project growth standards, the Intergrowth 21^st^ Project newborn charts were used to derive the birth weight z-scores and the Intergrowth 21^st^ Project postnatal growth charts were used to derive the z-scores at discharge.

Neonates were defined as SGA if their birthweight was below the 10^th^ percentile of the chart used. EUGR was defined as *cross-sectional-EUGR* if the weight was below the 10^th^ percentile at discharge, whereas *longitudinal-EUGR* if the weight loss was more than respectively 1 SDS (moderate) and 2 SDS (severe) between birth and discharge.

Pregnancy complications (intra-uterine growth restriction (IUGR), gestational diabetes, preeclampsia), antenatal steroid administration (complete prophylaxis-2 doses), and delivery mode; gender, gestational age at birth, Apgar score, morbidities related to prematurity (bronchopulmonary dysplasia, retinopathy of prematurity (ROP), late-onset sepsis (LOS), necrotizing enterocolitis), time (days) to regain birthweight, duration of parenteral nutrition (PN), days for achieving 100 ml/kg/day of enteral feeding and feeding with maternal milk were all recorded.

Bronchopulmonary dysplasia or chronic lung disease (BPD/CLD) was defined as moderate/severe according to Jobe’s classification [13] if there was need for oxygen at week 36 post menstrual age (PMA) if born gestational age (GA) <32 week and at >28 days postnatal age if born at GA ≥32 week (moderate CLD <30% oxygen, severe CLD ≥30% oxygen and/or positive pressure). Necrotizing enterocolitis (NEC) was defined as grade 2 or 3 according to modified Bell’s staging [14]; ROP was defined according to the international classification of ROP: any stage and severe ROP including stage 3 or more and/or plus disease were considered [15]. LOS was defined by a positive blood culture associated with clinical signs.

For the analysis of EUGR risk factors in our population, we used the *longitudinal-EUGR* definition since it seems to better predict the auxological long-term outcome [4] and psychomotor development at 18–24 months [16,17]. Moreover, it limits the impact of IUGR on EUGR diagnosis, since SGA babies have a high probability to be discharged <10°centile, despite of an adequate postnatal growth [11]. Weight loss of 1 SD was chosen as a cut-off, as the prevalence of 2 SD loss was low in our group. For this secondary analysis, INeS charts were chosen as reference, as they are the ones commonly used in our NICU clinical practice.

Descriptive analysis was conducted calculating frequency and percentage for categorical variables and median and Interquartile Range (IQR) for continuous variables.

We evaluated the percentage of SGA as the ratio between the number of children whose birthweight was below the 10^th^ percentile of the chart used and the total of newborns. We calculated, also, the EUGR percentage using the three definitions above mentioned: number of children with 1) weight below the 10^th^ percentile on discharge (*cross-sectional-EUGR)*, 2) weight loss > 1 SDS (*longitudinal-EUGR-1)*, and 3) weight loss > 2 SDS between birth and discharge (*longitudinal-EUGR-2)*, on the total of newborns, for all charts used.

Chi square test or exact Fisher test were used to evaluate the association between two categorical variables while Wilcoxon Mann Whitney non parametric test was applied to verify the difference in the distribution of a continuous variable between the categories of a dichotomous variable.

To identify EUGR risk factors, we used a backward selection logistic model adjusted for SGA at birth, sex and time to regain birthweight. Clinical data and anthropometric measures, both maternal and neonatal, were included as independent variables. A p-value<0.05 was considered as statistically significant. All statistical analysis was performed using SAS software, Version 9.4 (SAS Institute Inc., Cary, NC, USA).

Considering α = 0.05 and presuming EUGR prevalence at discharge of 72%, according to longitudinal EUGR definition and using INeS charts [4], with a margin of error of 6%, the sample size should be 216 infants.

## Results

Between 2009 and 2018, 266 VLBW infant were admitted to our NICU. Thirty-eight newborns were excluded for the following reasons: 24 died, 4 were transferred to other hospitals before 33 weeks of postmenstrual age, 9 had chromosomal abnormalities or major congenital abnormalities and 1 had no available data.

Hence, 228 VLBW were included in the study.

The percentage of SGA in our population did not change significantly according to the three different growth charts used. When assessing EUGR according to *cross-sectional-EUGR* and *longitudinal-EUGR-1* definitions, both INeS and Fenton charts estimated a statistically significantly higher prevalence of EUGR when compared to Intergrowth charts. On the contrary, EUGR prevalence when defined as *longitudinal-EUGR-2* did not significantly change according to reference growth chart (Table 1).

**Table 1:** Distribution of SGA and EUGR according to different reference growth charts in our VLBW population.

	Fenton	INeS	Intergrowth-21	p-value*
**SGA (%)**	22.4	22.8	28.2	0.27
**EUGR (%)**				
Cross-sectional EUGR (weight at discharge <10°ct)	33.5	40.9	23.8	**0.001**
Longitudinal-EUGR-1 (Loss of z-score > 1)	15	20.4	4	**<0.001**
Longitudinal-EUGR-2 (Loss of z-score > 2)	0.9	1.3	0.4	0.6

Chi -square test.

3 patients were discharged after week 42 and could not have been analysed with INeS charts, 1 patient was discharged after week 65 and could not have been analysed with Fenton and Intergrowth charts, 1 patient was born at week 23 and could not have been analysed with Intergrowth charts.

Abbreviations: SGA: Small for gestational age EUGR: Extrauterine growth restriction.

In our study 20 babies out of 228 were back transferred to local hospitals between 33+4 and 35+6 weeks postmenstrual age. The prevalence of EUGR in this group was lower than in the whole cohort (S1 Table).

EUGR infants were more likely to be male, and to have a significantly lower median birth weight and gestational age compared to their non-EUGR counterparts. Moreover, they had lower median APGAR score at 5-minute, they experienced a higher duration of parenteral nutrition PN (24 vs 16 days), took a higher median time to reach 100 ml/kg/day of enteral feeds and to regain birthweight. Some morbidities were significantly higher in the EUGR VLBW including LOS and ROP, while there were no differences regarding NEC and BPD (Table 2).

**Table 2:** Study population characteristics according to EUGR status (defined as loss of > 1 SDS and according to INeS charts).

	Total sample (n = 228) **	non-EUGR (n = 179)	EUGR* (n = 46)	p-value
**Mother characteristics**				
Steroid coverage—n (%)	188 (82.8)	149 (83.7)	37 (80.4)	0.60
Gestational DM—n (%)	23 (10.1)	18 (10.1)	4 (8.7)	0.77
IUGR—n (%)	72 (31.7)	64 (36.0)	8 (17.4)	**0.02**
Preeclampsia—n (%)	36 (15.9)	34 (19.1)	2 (4.3)	**0.02**
Delivery mode—n (%)				
Vaginal delivery	51 (22.4)	33 (18.4)	17 (37.0)	**0.01**
Caesarean section	177 (77.6)	146 (81.6)	29 (63.0)	
**Newborn characteristics**				
Male sex—n (%)	111 (48.7)	79 (44.1)	29 (63.0)	**0.02**
Singletons—n (%)	93 (40.8)	74 (41.3)	17 (37.0)	0.84
Gestational age (weeks)–median (IQR)	29 (28–31)	30 (28–31)	28 (26–29)	**<.0001**
Birthweight (g)–median (IQR)	1169 (919–1371)	1198 (968–1388)	940 (795–1368)	**0.04**
Weeks of hospitalization	8 (6–10)	7 (5–9)	10 (8–12)	**<.0001**
PMA at discharge (weeks)–median (IQR)	37 (36–38)	37 (36–38)	38 (37–39)	**0.001**
Weight at discharge (g)–median (IQR)	2615 (2280–2880)	2580 (2240–2880)	2665 (2350–2760)	0.52
APGAR score at 5 min–median (IQR)	8 (8–9)	9 (8–9)	8 (6–9)	**0.003**
LOS—n (%)	22 (9.7)	11 (6.1)	9 (19.6)	**0.004**
BPD—n (%)	22 (9.7)	14 (7.8)	6 (13.0)	0.27
NEC—n (%)	4 (1.8)	3 (1.7)	1 (2.2)	1.00
ROP—n (%)	47 (20.6)	29 (16.2)	16 (34.8)	**0.01**
Severe ROP	14 (6.2)	7 (3.9)	6 (13.0)	**0.02**
Duration of PN–median (IQR)	16.5 (12–24)	16 (12–20)	24 (16–36)	**<.0001**
Time to reach 100 ml/kg per os–median (IQR)	12 (9–17)	12 (9–16)	16 (12–27)	**0.0002**
Time to reach birthweight (days)–median (IQR)	9 (8–10)	8 (7–10)	10 (8–11)	**0.003**
Mother’s own milk during hospitalization—n (%)				
0% - 50%	67 (31.6)	54 (32.3)	13 (28.9)	0.66
>50%	145 (68.4)	113 (67.7)	32 (71.1)	

* EUGR defined as loss of > 1 SDS according to INeS charts.

** 3infants were excluded from the analysis, because discharged after week 42.

In the multivariate analysis, independently of birthweight, gender and time to regain birthweight, the risk of being EUGR was higher, although not significantly, if VLBW experienced a LOS (OR: 2.28–95%CI: 0.67–7.79), and ROP (OR 2.36–95%CI 0.94–5.94). Longer time to reach 100 ml/kg/day of enteral feeding was significantly associated with longitudinal EUGR (OR: 1.18–95%CI: 1.04–1.34). On the contrary, in our study having a mother who suffered from preeclampsia significantly reduced the risk of EUGR at discharge (Table 3).

**Table 3:** Odds Ratio (OR) and relative 95% Confidence interval (95%CI) of univariate and multivariate logistic regression.

Variable	Categories	EUGR (Loss of > 1 SDS–INeS)							
		Univariate*				Multivariate*			
		AIC	OR	95%CI		AIC	OR	95%CI	
**Preeclampsia**	**YES vs NO**	209.67	**0.28**	**0.06**	**1.24**	192.81	**0.15**	**0.03**	**0.78**
**ROP**	**YES vs NO**	207.23	**2.78**	**1.26**	**6.12**		**2.36**	**0.94**	**5.94**
**Late onset sepsis**	**YES vs NO**	204.91	**4.89**	**1.72**	**13.91**		**2.28**	**0.67**	**7.79**
**Time to reach 100 ml/kg per os**	**Units = 2**	197.362	**1.23**	**1.11**	**1.37**		**1.18**	**1.04**	**1.29**

*The models were adjusted by SGA at birth, sex and time to regain birthweight (days).

## Discussion

In our study we found that rates of EUGR varied significantly according to charts and definitions used, resulting higher when using INeS charts both when applying a *cross-sectional* and a *longitudinal-EUGR-1 definition*. At multivariate analyses, LOS and a longer time for reaching 100 ml/kg/day enteral feeding were associated with a higher risk of EUGR at discharge, while maternal preeclampsia reduced this risk.

In line with other studies, in our population frequency of SGA was higher, though not significantly so, when using the Intergrowth-21^st^ growth charts [9,10,18]. This result is expected, as Intergrowth-21 charts were plotted with a standard population, with the lowest risk factors known to affect prenatal and postnatal growth (low-risk women, non-smokers, with a normal pregnancy history with normal growing fetuses) and this may explain a tendency to overestimate SGA newborns.

On the contrary, when using Intergrowth-21th charts, EUGR prevalence was lower when compared to Fenton and INeS charts, both with the cross-sectional and the longitudinal definition. This result is consistent with previous studies [9,10,18–20] and this difference may be expected, as the Intergrowth charts are based on longitudinal growth of preterm babies, who grow in a completely different environment and with different metabolic responses compared to fetuses in the intrauterine environment. The difference between Intergrowth-21 and INeS charts is even more significant than between Intergrowth and Fenton and this could reflect the more homogeneous population that were used to design them.

Ideal growth for preterm babies is far from being clearly defined. One of the widely accepted goals of neonatal nutritional care is to try to replicate the intrauterine growth as close as possible [21] and, for this reason, the most commonly used growth charts have been the cross-sectional charts based on in utero growth. However, it is still debated whether it is appropriate to expect preterm babies to grow at the same rate as those in utero [22]. Benefits of improved growth on neurodevelopmental outcomes and chronic lung disease are well known, but the optimal pattern of growth in preterm infants to achieve good long-term health should also take into account the potential impact of excessive growth, associated with the risk of metabolic and cardiovascular disease in later life. The Intergrowth 21th Project therefore developed new growth standards from a cohort of uncomplicated pregnancies with normal growing fetuses, uncomplicated postnatal period and up-to-date nutritional support [8], and explain why EUGR prevalence–regardless of its cross-sectional or longitudinal definition–turns to be lower when compared to other reference charts.

These trends to lower rates of EUGR when using Intergrowth-21 charts have been reported by several other studies, both for cross sectional EUGR definition [9,10] and longitudinal EUGR [18]. In a European multicountry cohort, cross-sectional EUGR rates were reduced from 24% (Sweden) and 60% (Portugal) when using Fenton charts to 13% (Sweden) and 43% (Portugal), when using Intergrowth-21 [23].

Our study was done because knowing and monitoring the prevalence of EUGR in our Unit, is considered to be a quality measure of care for preterm infants [3]. In our population EUGR prevalence ranged from 40.9% using cross-sectional definition with INeS charts to 4% using longitudinal-1 definition with Intergrowth-21 standards. However, to draw comparisons between different centers is difficult not only because definition of EUGR and reference growth charts differ, but also because maternal and clinical characteristics of the study population, time at EUGR evaluation (36 weeks, 40 weeks PMA, discharge) and choice of ΔSDS threshold when applying the *longitudinal-EUGR* definition (> 1, >2 SDS), changes. EUGR rates from NICUs of high-income countries similar to ours, evaluated at discharge, varied from 17% to 77.2% for the *cross-sectional* EUGR [4,24–26], from 29.8–39.1% for *longitudinal-1* definition [27,28] and from 5.2–13% for *longitudinal-2 EUGR* [27–29].

When looking for risk factors, we confirmed that the characteristics of the study population are determinant to EUGR at discharge. The degree of longitudinal EUGR is influenced by birth weight z score: the lower the birthweight centile, the lower the probability to lose 1 or 2 SDS [18,28,30]. In our population male sex was also significantly related with poor growth, as reported in other studies [18,31,32] Therefore, we decided to adjust our logistic regression for low z-score at birth (SGA) and male sex. Physiological weight loss after birth can be a confounder in evaluating postnatal growth in preterm infants. In fact more recently some EUGR definitions consider the weight after 2 or 3 weeks from birth as the starting point for EUGR evaluation (post-loss longitudinal EUGR) [17,33]. In order to consider this aspect, we adjusted our multivariate analysis for ‘time to regain birth weight’.

Independently of the above factors, in our population we found that LOS and ROP more than doubled the risk for EUGR, although not statistically significant. In other studies [18,30,32,34] other comorbidities associated with prematurity had a significant effect on the incidence of growth restriction: patent ductus arteriosus, broncopulmonary dysplasia, necrotizing enterocolitis and late-onset sepsis, need for assisted ventilation, exposure to postnatal steroids and major brain lesions. These risk factors may only be markers for severity of illness: sick infants are often fed less than healthier infants, have increased metabolic demands, and their nutritional needs are rarely met, all of which result in malnutrition and poor growth. However, the presence of LOS as an independent risk factor underlines the necessity for implementing interventions targeted to reduce the incidence of neonatal sepsis [35]. The association between a slow postanatal weight gain and ROP is already known: in fact, poor postnatal growth is highly predictive of ROP and it is incorporated in several ROP prediction models [36].

In our analyses longer time to reach 100 ml/kg/day of enteral feeding was found to be a potential risk factor. Again, this may be an indirect sign of severity of illness, as increasing oral nutrition may be harder in sick infants. Intervention studies showed that optimising nutrition (such as introducing guidelines for increasing feeds and weaning PN) reduced the incidence of EUGR [37–39].

In our study it was surprising to find a negative association between preeclampsia and EUGR risk. As far as we know, no other study reported this association up to now. However, Gonzalez-Garcia et al showed that in a Spanish cohort of preterm babies, IUGR, which is known to be frequently associated with preeclampsia, was negatively associated with longitudinal EUGR [11]. This association may be explained by the fact that having both a low z-score at birth and losing >1 SD may be difficult to occur. Therefore, it is possible that the EUGR definition itself affects risk factors: a low z-score at birth may be positively associated with cross-sectional-EUGR, but negatively associated with longitudinal EUGR, as well as IUGR and preeclampsia [10,11,18].

Limitations of our study are inherent to the retrospective observational nature of the study. Our cohort is small and includes a wide range of gestational ages and birthweight categories. Another limitation may lie in the choice of discharge as a time point for assessing EUGR, as there is a wide range of time of evaluation and a long time passes between birth and discharge. Considering these two time points (birth and discharge) for evaluation of growth, we are not able to identify when loss of z-score occurred most often. As this study was conducted in a single medical centre, generalization of the data is limited. Moreover, residual confounding cannot be ruled out.

In conclusion, our study confirms a wide variability of EUGR rates when using different charts and definitions. It highlights the need to standardize criteria and the evaluation method for EUGR, which would facilitate comparisons between studies and help to improve nutrition in neonatal units and to perform studies on its long term implication.

## Supporting information

S1 Table Distribution of SGA and EUGR according to different growth charts in VLBW who were back transfered from our NICU to local hospitals between 33 and 35+6 weeks. (DOCX)
