# Supplementary material for: Extrauterine growth restriction in very low birth weight infants according to different growth charts: A retrospective 10 years observational study
Source: PLoS One. 2023 Apr 20;18(4):e0283367. doi: 10.1371/journal.pone.0283367 (PMC10118091; doi:10.1371/journal.pone.0283367)
Supplement: S1 Table — (DOCX) [file pone.0283367.s001.docx]

**S1 Table. Distribution of SGA and EUGR according to different growth charts in VLBW who were back transfered from our NICU to local hospitals between 33 and 35+6 weeks**

|  | Fenton | INeS | Intergrowth-21 |
| --- | --- | --- | --- |
| SGA (n, %) | 3 (15.0) | 2 (10.0) | 4 (20.0) |
| EUGR (n, %) |  | | |
| Cross-sectional EUGR (weight at discharge <10°ct) | 7 (35.0) | 6 (30.0) | 4 (20.0) |
| Longitudinal-EUGR-1 (Loss of z-score > 1 ) | 1 (5.0) | 2 (10.0) | 0 (0.0) |
| Longitudinal-EUGR-2 (Loss of z-score >2) | 0 (0.0) | 0 (0.0) | 0 (0.0) |
